# An assessment of fluoride related posts on Instagram

**DOI:** 10.15171/hpp.2019.11

**Published:** 2019-01-23

**Authors:** Corey H. Basch, Nicole Milano, Grace C. Hillyer

**Affiliations:** ^1^Department of Public Health, William Paterson University, Wayne, NJ 07470, USA; ^2^School of Social Work, Rutgers University, New Brunswick, NJ 08901, USA; ^3^Department of Epidemiology, Mailman School of Public Health, Columbia University NY, NY 10032, USA

**Keywords:** Fluoride, Social Media, Internet

## Abstract

**Background:** Social media is a driving force in the sharing of information. The purpose of this study is to describe fluoride related content on Instagram, a popular social media platform.

**Methods: ** Content categories were created and coded to better describe the nature of the posts.Data collection occurred in three sessions, two months apart. Only relevant posts that included images and had text written in the English language were included.

**Results: ** The most common topics were conspiracy theory, contained in 37.3% of posts, followed by dangers of fluoride to health (30.3%) and benefits of fluoride to teeth (28.7%). Of the posts reviewed, 96/300 (32.0%) contained pro-fluoride content while 139/300 (63.0%) posts featured anti-fluoride content. Content varied significantly between pro- and anti-fluoride posts.

**Conclusion:** Our review of Instagram posts revealed that there were approximately 300 posts focused on fluoride related content. Of these posts, there was a higher number of anti-fluoride related content compared to pro-fluoride related content. With accessibility comes the potential for misinformation. Future efforts from medical providers need to focus on educating consumers about reliable sources for health information on the internet.

## Introduction

Fluoride is a naturally occurring mineral, that is beneficial in preventing tooth decay as it strengthens the tooth’s enamel.^1^ While there is typically some fluoride in water, the amount is not significant enough to prevent decay, and therefore, state and local governments in the United States make decisions related to fluoridating public water supplies.^1^ Touted as one of the greatest public health achievements of the 20th century, community water fluoridation has played a significant role in preventing dental caries.^2^ Fluoride is also added to toothpaste and other products such as mouthwash. Despite the benefits, over-fluoridation can have negative impacts on teeth and health in general.^3,4^ Research has indicated that toothpaste advertisements often depict well above the recommended levels.^5,6^ Thus, consumers may be confused about the amount of fluoride that is safe to use or skeptical about the addition of this mineral to their products and water supply.

In addition to print advertisements, the internet has become a main source of information for consumers. Roughly 60 percent of adults in the United States have reported accessing the internet for health information.^7^ Parents making decisions about their children’s health are also drawn to the internet as a guide.^8^ Social media is a driving force in the sharing of information. Instagram, boasting an estimated 1 billion users as of June 2018 is a popular site for sharing images.^9^ There is a gap in the literature regarding fluoride related content on Instagram. Therefore, the purpose of this study is to describe fluoride related content on Instagram.

## Materials and Methods

This is a cross-sectional study. Prior to data collection, content categories were established using a Centers for Disease Control fact sheet as a guide^1^ and adding categories as necessary. A coding sheet was created using the categories and was pilot tested on 15 images that were derived using the hashtag #fluoride. Two researchers (CHB and a research assistant) pilot tested the coding sheet and had perfect agreement when coding a sample of images. The main data collection was conducted by CHB on three separate dates, which were spaced two months apart in 2018 (April 19, June 19, and August 19). At each juncture in time, 100 recent posts using the hashtag #fluoride were coded. Posts were printed on each respective data collection day and labeled with a description in the event that the post needed to be revisited. These were later destroyed and discarded. For each of the three data collection sessions, posts were sifted for relevance and only posts that included images and had text written in the English language were included. At the first data collection point, there were 57 127 posts and the second there were 60 038, and at the third there were 62 037. A total of 56 posts were excluded in the first round, 60 in the second and 63 in the third.

### Data analysis

Data were presented by frequency (percentage) for categorical variables, and mean, standard deviation and range for continuous variables (video views and likes). Univariable analysis comparing pro-fluoride vs. anti-fluoride posts were conducted using chi square test for categorical and dichotomous variables and Student’s *t* test for continuous variables. *P* values <0.05 were considered statistically significant. All analyses were performed using IBM SPSS Statistics for Windows, Version 25, IBM Corp. Released 2017, Armonk, NY: IBM Corp.

## Results

In total, 300 Instagram posts over the course of April, June and August 2018 were reviewed for their fluoride related content. The majority of posts displayed only an image (59.7%), had a mean of 645 [SD = 1990.7] views and 67.9 [SD = 310.3] likes (Table 1). The most common topics were conspiracy theory, contained in 37.3% of posts, followed by dangers of fluoride to health (30.3%) and benefits of fluoride to teeth (28.7%). Of the posts reviewed, 96/300 (32.0%) contained pro-fluoride content while 139/300 (63.0%) posts featured anti-fluoride content. Anti-fluoride posts saw an increase in June 2018 over the preceding and following months compared to the number of pro-fluoride posts (*P* = 0.01) and were over three times more likely than pro-fluoride posts (15.3% vs. 4.2%, *P* = 0.005) to display video content.

Content varied significantly between pro- vs. anti-fluoride posts. Anti-fluoride posts more often mentioned conspiracy theories (56.1% vs. 0.0%, *P *≤ 0.001), community fluoride programs (25.4% vs. 5.2%, *P *≤ 0.001) and dangers of fluoride to health (45.5% vs. 2.1%, *P *≤ 0.001) and teeth (18.5% vs. 2.1%, *P *≤ 0.001). Compared to anti-fluoride posts, pro-fluoride posts were more likely to discuss the benefits of fluoride to teeth. (88.5% vs. 0.5% *P *≤ 0.001) and promote a service (59.4% vs. 1.1%, *P *≤ 0.001). Pro-fluoride posts also featured promotion of a product almost three times as often (58.3% vs. 21.7%, *P *≤ 0.001) and twice as often discussed fluoridation of toothpaste and mouthwash (32.2% vs. 16.4%, *P *= 0.002) compared to anti-fluoride posts.

## Discussion

Our review of Instagram posts throughout the months of April, June and August revealed that there were approximately 300 posts focused on fluoride related content. Of these posts, there was a higher number of anti-fluoride related content compared to pro-fluoride related content. The anti-fluoride related posts featured conspiracy theories and the dangers of fluoride to health and teeth. The pro-fluoride posts focused on the benefits of fluoride to teeth yet these posts did not occur as frequently as anti-fluoride posts. Anti-fluoride content also saw more engagement than pro-fluoride posts. This means more individuals liked the posts or watched videos featuring anti-fluoride content.

Other studies have yielded similar results when evaluating the frequency of anti-fluoride content on social media.^10^ It has been found that peer to peer influence, as is common among social media, is a primary factor when shaping beliefs about health practices. Social media provides connectedness for those that hold similar beliefs and further encourages misconceptions about health.^11^ Furthermore, ample research suggests that there is a significant amount of misinformation related to health matters on social media that can lead to potentially dangerous health practices.

Additionally, medical providers, such as dentists, need to find a way to communicate the detrimental effects of fluoride refusal to parents in a way that actively engages the parent in care of their child. Anti-fluoride beliefs account for large percentages of new parents refusing fluoride from their pediatric dentists.^12^ As a result, this preventative care practice is not being implemented and children are exposed to more illnesses and avoidable suffering. It has been shown that when parents are told they “must get this fluoride” they are more likely to refuse.^11^ In contrast, asking parents to engage in their concerns about fluoride allows for debunking of misconceptions found from unreliable sources.

The limitations of this study include the cross-sectional design, the short time period in which posts were evaluated and the small number of posted evaluated. Additionally, only posts in English were evaluated. It remains unclear how individuals that do not speak English as their primary language are affected by fluoride posts. Future research can focus on fluoridation in different areas of the world. Despite these limitations, our results suggest that information about fluoride content is engaging to the public and could be an outlet for positive health information in the future.

Social media applications such as Instagram have the ability to communicate health beliefs in an easy and free method. With this accessibility, however, comes the potential for misinformation. Future efforts from medical providers need to focus on educating consumers about reliable sources for health information on the internet.

## Ethical approval

The Institutional Review Board at William Paterson University does not review studies that do not involve human subjects.

## Competing interests

The authors declare that they have no competing interests.

## Funding

Funding for this study was made possible by the Center for Research at William Paterson University.

## Authors’ contributions

CHB conceptualized the study, designed the methodology and collected the data. NM and GCH conducted data analysis. CHB, NM, and GCH participated in all phases of manuscript development.

## Acknowledgments

We would like to thank Rachelle Romero, for participating in pilot phase of this study.

**Table 1:** Characteristics and content of Instagram postings in April, June, and August, 2018 related to fluoride (N = 300)

	**Total** **N = 300**	**Pro-fluoride** **(n = 96, 32.0%)**	**Anti-fluoride** **(n = 189, 63.0%)**	***P*** ** value**
**Characteristics**				
Month				0.01
April	100 (33.3)	38 (38.4)	61 (61.6)	
June	100 (33.3)	20 (21.5)	73 (78.5)	
August	100 (33.3)	38 (40.9)	55 (59.1)	
Format				0.038
Text only	21 (7.0)	6 (6.3)	15 (7.9)	
Text and image	64 (21.3)	24 (25.0)	39 (20.6)	
Image only	179 (59.7)	62 (64.6)	106 (56.1)	
Video	36 (12.0)	4 (4.2)	29 (15.3)	0.005
Range Video Views	24-11,050	59-204	24-11,050	
Mean Views [SD]	645 [1990.7]	128.7 [70.2]	748.8 [2211.5]	0.58
Instagram Posts w/ “Likes”	239 (79.7)	82 (85.4)	147 (77.8)	0.12
Range “Likes”	1-4111	1-264	1-4111	
Mean “Likes” [SD]	67.9 [310.3]	32.6 [45.7]	89.5 [393.1]	0.19
**Topics of Instagram posts**				
Conspiracy theory				<0.001
Yes	112 (37.3)	0 (0.0)	106 (56.1)	
No	188 (62.7)	96 (100.0)	83 (43.9)	
Community fluoride program				<0.001
Yes	55 (18.3)	5 (5.2)	48 (25.4)	
No	245 (81.7)	91 (94.8)	141 (74.8)	
Dangers of fluoride to health				<0.001
Yes	91 (30.3)	2 (2.1)	86 (45.5)	
No	209 (69.7)	94 (97.9)	103 (54.5)	
Dangers of fluoride to teeth				<0.001
Yes	38 (12.7)	2 (2.1)	35 (18.5)	
No	262 (87.3)	94 (97.9)	154 (81.5)	
Benefits of fluoride to teeth				<0.001
Yes	86 (28.7)	85 (88.5)	1 (0.5)	
No	214 (71.3)	11 (11.5)	188 (99.5)	
Promotes a service				<0.001
Yes	61 (20.3)	57 (59.4)	2 (1.1)	
No	239 (79.7)	39 (40.6)	187 (98.9)	
Promotes a product				<0.001
Yes	102 (24.0)	56 (58.3)	41 (21.7)	
No	198 (66.0)	40 (41.7)	148 (78.3)	
Fluoridation of toothpaste/mouthwash				0.002
Yes	65 (21.7)	31 (32.3)	31 (16.4)	
No	235 (78.3)	65 (67.7)	158 (83.6)	
Benefits of fluoride in water				0.001
Yes	6 (2.0)	6 (6.3)	0 (0.0)	
No	294 (98.0)	90 (93.8)	189 (100.0)	
Removing fluoride from body				0.55
Yes	4 (1.3)	0 (0.0)	3 (1.6)	
No	296 (98.7)	96 (100.0)	186 (98.4)	
